# Comparison of eTEP and IPOM for ventral hernia surgery in the early postoperative period: a retrospective cohort study of a tertiary university centre

**DOI:** 10.1007/s10029-024-03125-6

**Published:** 2024-09-16

**Authors:** Lukas Wieland, Fadl Alfarawan, Maximilian Bockhorn, Nader El-Sourani

**Affiliations:** 1 Department for General and Visceral Surgery, University Hospital Oldenburg Klinikum Oldenburg AöR, Rahel-Straus-Straße 10, Oldenburg, 26133 Germany; 2 Carl von Ossietzky Universität Oldenburg Fakultät VI - Medizin und Gesundheitswissenschaften, Ammerländer Heerstraße 114-118, Oldenburg, 26129 Germany

**Keywords:** eTEP, IPOM, Ventral hernias, Extraperitoneal

## Abstract

**Purpose:**

The extended totally extraperitoneal technique (eTEP) is a relatively new laparoscopic approach to address ventral hernias. Since this technique is not widely used yet, the literature regarding its efficacy and safety is limited, especially when compared to more established surgical techniques like intraperitoneal onlay mesh (IPOM). This study aimed at contributing to the expanding body of evidence for eTEP, by comparing the early outcomes of eTEP and IPOM surgeries for ventral hernias.

**Methods:**

This monocentric, retrospective cohort study compared patients with ventral hernias that were treated with eTEP or IPOM from 2019 to 2023.

**Results:**

A total of 123 patients were analysed. 92 underwent eTEP and 31 IPOM respectively. Both groups were overall comparable. The IPOM group had a higher proportion of incisional hernias (61,29% vs. 21,74%, *p* < 0,001). This was taken into account for in a subgroup analysis of only primary hernias. The IPOM group had a significantly longer admission time (eTEP: 3 days, IPOM: 4 days, *p* < 0,001). The subgroup analysis revealed a statistically significant shorter surgery time in IPOM (median of 66,5 min vs. 106,5 min; *p* = 0,043) and a lower rate of postoperative complications in eTEP (eTEP: 4,17%, IPOM: 25%. *p* = 0,009). The eTEP group reported lower postoperative pain, yet without statistical significance.

**Conclusion:**

eTEP for ventral hernia repair appears to be non-inferior to IPOM. Compared to IPOM it leads to shorter postoperative hospital stay and a potentially lower complication rate, despite a longer operation time.

## Introduction

Ventral hernias are a common condition and affect up to 20% of all adults [1]. They impose a significant burden by reducing quality of life and potentially leading to further complications, like incarceration or strangulation, which can result in higher mortality rates compared to elective cases [2–4].

Surgical interventions for ventral hernia repair are common, with around 350.000 procedures carried out annually in the United States alone [3]. Various surgical techniques exist, with both laparoscopic and open approaches. Almost all primary ventral hernia repairs (excluding hernias < 1 cm in which primary repair could be considered) and all incisional hernia repairs require the placement of a reinforcement mesh to reduce tissue tension, thus dramatically reducing recurrence rates when compared to primary repairs [5, 6]. It is well-established that the sublay position of a mesh is the superior anatomical position, not only due to less need of fixation but also because it avoids contact with abdominal viscera, resulting in fewer long-term complications. It also has less SSO and SSI when compared to the onlay position [3, 7–10].

Since 1990, laparoscopic procedures have gained popularity because they were linked to a lower complication rate, shorter hospital stay and quicker return to work [11]. The extended totally extraperitoneal repair (eTEP) is a relatively new minimal invasive surgical procedure that utilises an artificially created space between the rectus abdominis and posterior rectus sheath for mesh insertion. It is a promising technique, as it combines the established advantages of the laparoscopic approach, and the placement of the mesh in the retrorectus space. Furthermore, because the procedure occurs outside the peritoneum, it theoretically reduces risks of complications like intraperitoneal adhesions [10, 12]. Originally described for laparoscopic inguinal hernia repair, eTEP has since been adapted for ventral and incisional hernias. [13]. Previously, IPOM was widely considered the standard laparoscopic procedure for ventral hernias [14].

Due to its novelty, scientific evidence for eTEP compared to other surgical techniques is limited. Some studies suggest that eTEP has distinct advantages over IPOM, primarily arising from the aforementioned advantages of using the retrorectus space for mesh implantation. Theoretical advantages of eTEP include lower postoperative pain levels, shorter admission periods and fewer overall postoperative complications and infections [5, 6, 10, 15]. The majority of the studies that compared the two treatment options had relatively small sample sizes, particularly in the eTEP groups, where sample sizes ranged from 27 to 46 [10, 15–17]. Another limitation of the aforementioned studies is that only one other study [15] performed a subgroup analysis. Incisional hernias, are typically more challenging surgeries with higher complication rates and longer hospital admissions, and could confound the outcomes if analysed together with primary hernias [18, 19]. To account for this limitation and we conducted a subgroup analysis focusing solely on primary hernias.

Our study aims at providing further insights into the advantages and disadvantages of eTEP and contributing to the expanding body of evidence on the efficacy, safety and short-term outcomes of eTEP in comparison to IPOM, particularly addressing the above-mentioned limitations. Due to our relatively large sample size and sub-group analysis distinguishing between incisional and ventral hernias we intend to offer more robust results to the current body of evidence. Comparing eTEP with IPOM was considered the most appropriate since both techniques are performed minimally invasive, making them more comparable than open procedures like the open sublay technique, especially in regards of short-term outcomes.

## Materials and methods

A monocentric, retrospective cohort study was conducted at the Department of General and Visceral Surgery at the Klinikum Oldenburg AöR. The study included patients with ventral hernias who underwent treatment with either IPOM or eTEP between 2019 and 2023. All eTEP procedures in that period were performed by the same surgeon. The data were gathered retrospectively from the medical records and added to a database. All patients received a follow-up appointment, scheduled 30 days postoperative. If patients had any complications beforehand, an earlier follow-up was scheduled. Following data collection, a comparative analysis of the short-term outcomes of the two procedures was performed.

Initially, all patients presenting with primary M2-M4 hernias and a defect size between 2 and 7 cm diameter were eligible for eTEP. For smaller defect sizes, eTEP was performed only in combination with significant rectus diastasis. With growing expertise in this field, the criteria were expanded to also include patients with umbilical hernias extending into the M1 or M5 area, as well as patients with incisional hernias. In these cases, a CT scan performed to plan the optimal surgical strategy. Carbonell’s algorithm was used to determine whether patients additionally required a transversus abdominis release (TAR) [20].

Absolute contraindications for performing eTEP included hernia types with defect sizes greater than 7 cm, prior sublay mesh implantations and isolated subxiphoid incisional hernias.

Contraindications for IPOM included defect sizes over 7 cm, severe adhesions after multiple laparotomies, burst abdomen, Crohn’s disease, gastrointestinal fistula, as well as M1, M5, and L3-L4 hernias.

The patients gave written consent that their data would be used for research and the universities ethics commission approved of this study.

### Surgical technique

#### eTEP

**Fig. 1:**
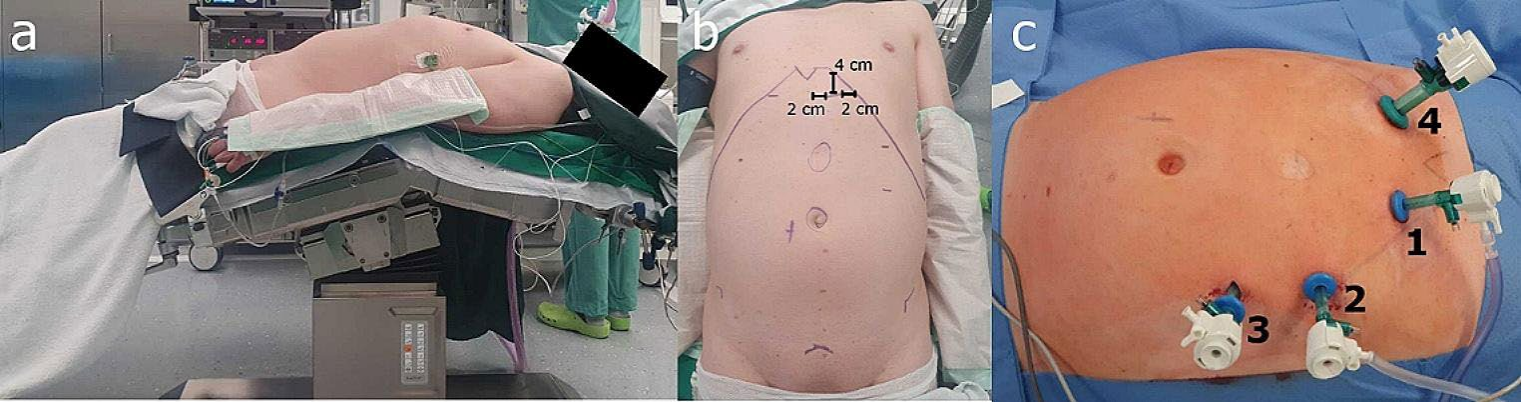
**(a)** Positioning of a patient; **(b)** Preoperative markings; **(c)** Trocars labelled according to order of introduction

The patient lays on the operating table in supine position. The arms are positioned alongside the body, slightly retroflexed, in order to increase the space between the rib margin and the anterior superior iliac crest (Fig. 11). A scheme of the costal cartilage, hernia and planned locations for the trocars is drawn on the patients abdomen (Fig. 1b). The arrangement of ports depends on the site of the defect and follows the principles of the eTEP procedure in which the retromuscular space is dissected on one side before progressing to the contralateral side. The surgeon is assisted by two monitors, one placed at the head of the patient and the other at the feet.

**Fig. 2:**
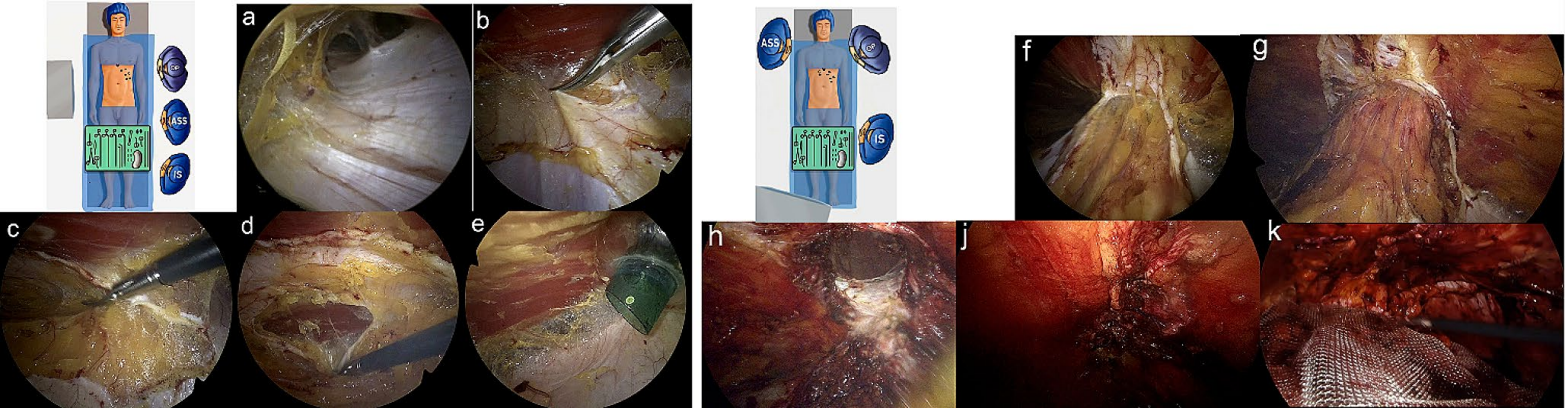
**(a)** Blunt dissection in the retromuscular space; **(b)** Identification and transection of the posterior fascial layer on the left side; **(c)** Preperitoneal dissection behind the linea alba; **(d)** Visualisation of the contralateral posterior fascial layer and transection; opening of the right-sided retromuscular space; **(e)** Placement of a camera trocar in the right retro-muscular space; **(f)** Cranio-caudal view depicting both retro-muscular spaces as well as the preperitoneal space; **(g)** Cranial depiction of the hernia defect; **(h)** Repositioning of the hernia sac along with its content; **(j)** Continuous closure of the hernia defect **(k)** Placement of the mesh

To prepare the trocar insertion, an approximately 12 mm wide incision is made 4 cm subcostal and half way between midline and costal cartilage. The rectus muscle is identified and incised, followed by expansion of the space between the rectus and the posterior fascia. Subsequently, a 12 mm optical trocar is inserted. The space is inflated with carbon dioxide and further dissected (Fig. 2a). A 10 mm, 30° laparoscope is then introduced to explore the retromuscular space. Afterwards, two additional 10 mm ports are inserted caudal to the optical trocar (Fig. 1c).

Dissection with diathermy coagulation continues until the medial margin of the rectus abdominis’ posterior sheath is reached. The neurovascular bundle at the semilunar line marks the lateral boundary for the dissection. The afore identified posterior fascial sheath is incised (Fig. 2b), followed by dissection of the preperitoneal space behind the linea alba (Fig. 2c) and opening of the contralateral posterior fascial layer (Fig. 2d), resulting in a single retromuscular plane. Afterwards, another optical trocar is inserted in the contralateral retro muscular space for further visualisation (Fig. 2e).

The reconstructive surgery involves suturing the linea alba with an absorbable 0 Stratafix^®^ suture, as well as repositioning and closing the hernial defects with a continuous absorbable self-anchoring 2 − 0 Stratafix^®^ suture (Fig. 2g-j). Reconstruction of the posterior plane is then carried out to form a retromuscular pocket, providing space for a polypropylene Bard^®^ softmesh with a 5 cm overlap in all directions. Subsequently, the mesh is positioned within the retromuscular box (Fig. 2k) and the retro muscular space is deflated under visualisation to ensure proper alignment of the mesh.

Depending on the size and complexity of the hernia, a TAR was considered.

#### IPOM

The patient is positioned supine with arms bilaterally placed and sufficiently cushioned. A subcostal incision is made on the left side and a pneumoperitoneum is established by the means of a blunt 10 mm trocar. This trocar is inserted laterally in the mid-abdomen using the Visioport technique. Subsequently, a 30° optic is introduced.

Under visualisation, 5 mm trocars are positioned subcostal on the left side and in the lower left abdomen. To allow uncomplicated placement of the mesh around the hernia defect, the ligamentum falciformis hepatis is partial dissected from the abdominal wall.

The hernia defect is laparoscopically closed, using 0 Stratafix^®^ sutures. A Ventralight™ PS echo mesh, with a minimum of 5 cm overlap of the hernia defect, rolled up, and introduced through the trocar on the left abdominal wall. It is then carefully unrolled and positioned intraabdominally in front of the hernia defect. The mesh is secured in place by circularly placing absorbable Sorbafix^®^ anchors in two rows.

### Variables

The analysed data encompassed patient demographics, including age, sex, and Body Mass Index (BMI). Additionally, risk factors were reported that could lead to poorer outcomes, including Chronic Obstructive Pulmonary Disease (COPD), cardiovascular diseases, diabetes, smoking history, liver cirrhosis and obesity. The preoperative condition of the patients was described via the Society of Anaesthesiologists physical status classification system (ASA).

Hernias were categorized as primary or incisional and their locations was described according to the European Hernia Society (EHS) [21]. The defect size was reported in square centimetres. For patients presenting with hernias spanning multiple anatomical sites, each affected location was documented as separate hernia. Consequently, the total count of reported hernias exceeded the number of patients.

Surgical data included the date of the surgery, its duration, intraoperative complications, the conversion rate to another surgical approach, and the size of the implanted mesh.

Postoperative pain levels were measured at rest during the first three postoperative days, using the Visual Analog Scale (VAS). The VAS was measured at least three times a day to account for considering temporary pain relief from as-needed medication. The average VAS for each day was used for data analysis. All patients received analgesia following our institutional pain management protocol, including a baseline treatment of metamizol, 1000 mg, administered orally, 4–6 times daily. If the VAS exceeded 4, oral oxycodone/naloxone, 5–10 mg twice daily, was added to the baseline regimen. For acute pain attacks, oral morphine, 10 mg, was available to patients.

The total admission period was reported, as well as postoperative complications along with their respective Clavien-Dindo classification. In addition to general complications, wound-specific complications were categorised as surgical site infection (SSI), surgical site occurrence (SSO) and surgical site occurrence requiring procedural intervention (SSOPI) [22]. All infections, both superficial and deep, were classified as SSI. SSOs described non-infective surgical site-specific complications, including haematoma, seroma, wound drainage, delayed wound healing, wound dehiscence or reopening, necrosis and early recurrences. Any SSI or SSO which required an invasive intervention was additionally classified as SSOPI.

### Statistical analysis

Statistical data analysis was performed using SPSS version 29. For continuous variables, a histogram was created to evaluate the data distribution. If the distribution appeared normal, the variable was reported as mean and standard deviation, otherwise as median and interquartile range. If the histogram was inconclusive a Shapiro-Wilk-Test was performed to assess normality. The dataset was divided into an eTEP and an IPOM group.

For the comparison of the groups, an unpaired t-test was used for normally distributed continuous variables. If the distribution was not normal, a Mann-Whitney U Test was used instead. Categorical variables were analysed with a chi-square test. A p-value < 0,05 was considered significant. If data was missing, the respective variables were deleted pairwise.

To minimise potential bias and to ensure a more homogeneous patient group, a subgroup analysis of only primary hernias was conducted, by using the same statistical methods. To further reduce potential confounders, an attempt was made to perform propensity score matching. However, due to the limited size of this dataset, no adequate matches could be achieved, with average calliper distances significantly exceeding the generally acceptable calliper of 0,2 [23].

### Patient characteristics

Between January 2019 and March 2023, a total of 123 patients underwent ventral hernia surgery at our centre. Of these, 92 were treated with the eTEP procedure and 31 with IPOM. Initially, 94 surgeries were started as eTEP procedures, however two of them were converted to IPOM during surgery. For the purposes of data analysis, these cases were classified as IPOM patients.

The mean age and BMI were comparable between both groups (Age: eTEP: 51,82 years [range: 18–84], IPOM: 53,81 [range:31–92], *p* = 0,493; BMI: eTEP: 32,31 kg/m² [range: 21–57], IPOM: 31,84 kg/m² [range: 22–55], *p* = 0,646).

There was no significant difference in gender distribution.

Median defect sizes were comparable between the two groups (eTEP: 6 cm² [range: 1-200], IPOM: 8 cm² [1-270], *p* = 0,894).

The eTEP group had a higher fraction of ASA II scores (eTEP: 67,39%, IPOM: 41,94%, *p* = 0,012) and a lower fraction of ASA III scores (eTEP: 26,09%), IPOM: 48,39%, *p* = 0,021)

A significantly larger proportion of incisional hernias was observed in the IPOM group compared to the eTEP group (eTEP: 21,74%, IPOM: 61,29%, *p* < 0,001).

Patients with a positive smoking history were more common in the IPOM group (eTEP: 20,65%), IPOM: 38,71%, *p* = 0,045). No significant differences were detected for other risk factors. Patient demographics are shown in Table 1.

**Table 1:** Total group - demographics

Variables	eTEP (*n* = 92)	IPOM (*n* = 31)	*p*-value
Age (years)	51,82 (± 13,15)*	53,81 (± 16,15)*	0,493
BMI (kg/cm^2^)	32,31 (± 7,45)*	31,81 (± 7,17)*	0,646
Sex			0,151
Male (%)	58 (63,04%)	15 (48,39%)	
Female (%)	34 (36,96%)	16 (51,61%)	
ASA score			
I	6 (6,52%)	3 (9,68%)	0,56
II	62 (67,39%)	13 (41,94%)	**0**,**012**
III	24 (26,09%)	15 (48,39%)	**0**,**021**
IV	0 (0%)	0 (0%)	/
V	0 (0%)	0 (0%)	/
Risk factors			
COPD (%)	2 (2,17%)	2 (6,45%)	0,246
Cardiovascular diseases (%)	48 (52,17%)	15 (48,39%)	0,715
Diabetes (%)	11 (11,96%)	3 (9,68%)	0,73
Smoking (%)	19 (20,65%)	12 (38,71%)	**0**,**045**
Liver cirrhosis (%)	0 (0%)	1 (3,23%)	0,085
Adipositas (%)	51 (55,43%)	17 (54,84%)	0,954
Defect size (cm2)	6 (IQR 9,25)†	8 (IQR 16,25)†	0,894
Hernia type			**< 0**,**001**
Incisional	20 (21,74%)	19 (61,29%)	
Primary	72 (78,26%)	12 (38,71)	

* = Mean + SD; † = Median + IQR; BMI = Body mass index; ASA = American Society of Anesthesiologists; COPD = Chronic obstructive pulmonary disease

### Hernia classifications

The most common hernia type were umbilical hernias (M3) in both groups, however the relative frequency of M3 hernias was higher in the eTEP group (eTEP: 57,48%, IPOM: 43,75%, *p* < 0,001).

No other hernia type showed significant differences in incidence between the two groups (Table 2).

**Table 2:** Total group - hernia types

European Hernia Society Classification	eTEP		IPOM		*p*-value
	*n* = 127	%	*n* = 32	%	
M1	2	1,57%	0	0%	0,408
M2	41	32,29%	13	40.63%	0,799
M3	73	57,48%	14	43.75%	**< 0**,**001**
M4	8	6,30%	4	12.5%	0,495
M5	3	2,36%	1	3.13%	0,992

### Surgical results

No intraoperative complications occurred in both groups.

The median surgery time was slightly shorter in the IPOM group, but the difference was not significant (eTEP: 110 min. [range: 49–290], IPOM: 88 min. [range: 44–235], *p* = 0,064).

**Fig. 3:**
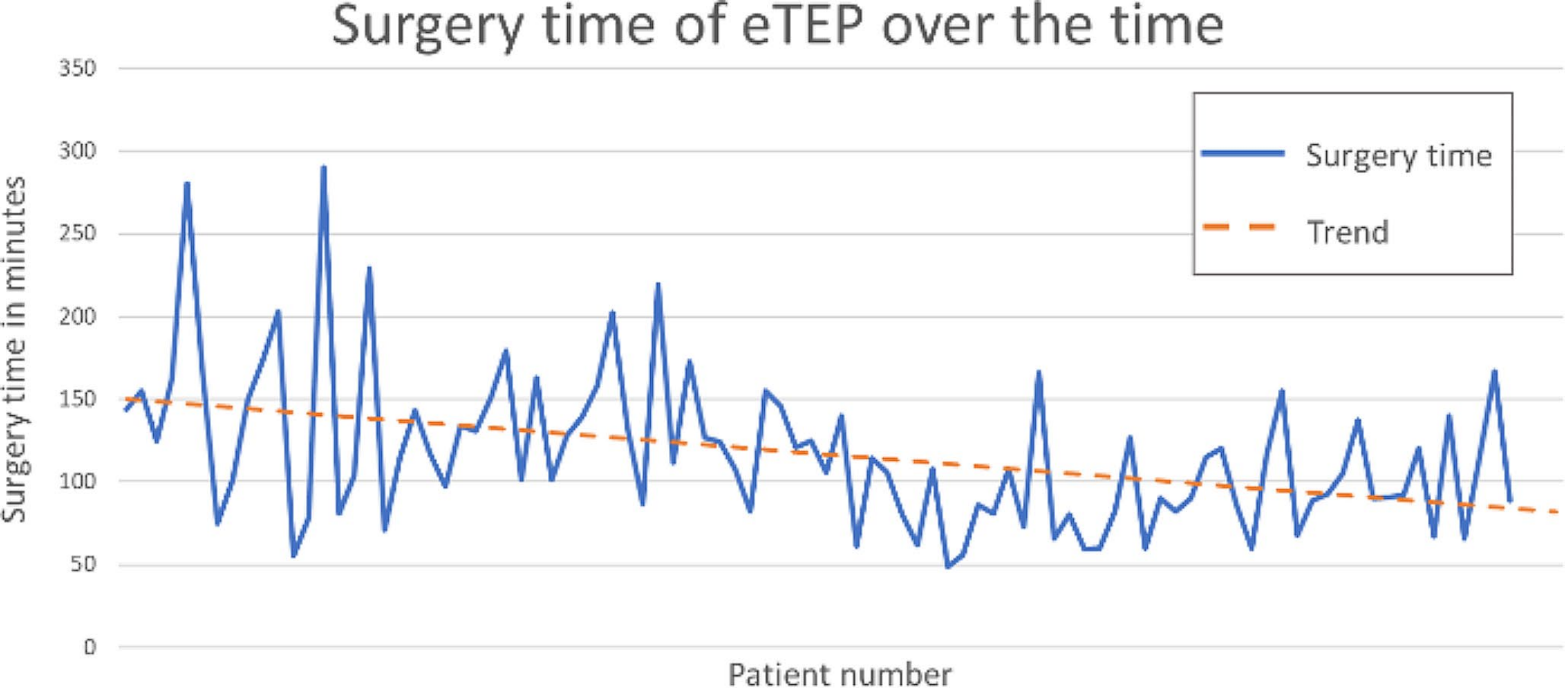
The duration of the individual eTEP surgeries and the trend over the course of time

Initially, a large variability in the duration of the individual eTEP surgeries was observed. However, with an increasing number of cases, in the variability of the surgery times narrowed. Furthermore, the trend showed an overall decrease in the surgery time (Fig. 3).

The median mesh size used in the eTEP group was significantly larger (eTEP: 450 cm² [range: 36–900], IPOM: 177 cm² [range: 79-1036], *p* < 0,001).

Out of 94 surgeries that were started as eTEP, two had to be converted to IPOM intraoperatively, due to adhesions and perforations of peritoneum resulting in an inadequate expansion of the retrorectus space. None of the IPOM surgeries had to be converted. The difference in the conversion rate was not significant (*p* = 0,428).

The intraoperative results are depicted in Table 3.

**Table 3:** Total group – outcomes

Variables	eTEP (*n* = 92)	IPOM (*n* = 31)	*p*-value
Mesh size (cm^2^)	450 (IQR 125)†	177 (IQR 187)†	**< 0**,**001**
Operation time (minutes)	110 (IQR 60)†	88 (IQR 80)†	0,064
Intraoperative complications ‡	0 (0%)	0 (0%)	/
Conversion Rate ‡	2 (2,13%)	0 (0%)	0,428
Postoperative complications	7 (7,61%)	7 (22,58%)	**0**,**023**
Clavien Dindo			0,15
I	2 (28,57%)	3 (42,86%)	
II	1 (14,29%)	1 (14,29%)	
IIIa	1 (14,29%)	0 (0%)	
IIIb	3 (42,86%)	3 (42,86%)	
IV	0 (0%)	0 (0%)	
V	0 (0%)	0 (0%)	
Admission period (days)	3 (IQR 1)†	4 (IQR 1)†	**< 0.001**
VAS			
Postoperative day 1	1,4 (IQR 1,92)†	1,88 (IQR 2,1)†	0,436
Postoperative day 2	1 (IQR 2)†	2 (IQR 3)†	0,144
Postoperative day 3	0,5 (IQR 2)†	0,8 (IQR 2)†	0,929

* = Mean + SD; † = Median + IQR; ‡ = 94 cases were started as eTEP initially, but converted to IPOM intraoperative; VAS = Visual Analogue Scale

### Postoperative results

We observed a significant higher postoperative complication rate in the patient group that underwent IPOM (eTEP: 7,61%, IPOM: 22,58%, *p* = 0,023).

Postoperative complications were observed in 7 cases among both groups as seen in Table 4.

**Table 4:** Postoperative complications

Complication	*n*	Description
eTEP		
Postoperative bleeding	1	Bleeding in the retrorectus space, requiring transfusion of two units of packed cells
Periumbilical abscess	1	Developed two weeks post-surgery; treated with incision and vacuum-assisted closure (VAC)
Hernia recurrence	1	Occurred during postoperative admission; required another operation using the sublay technique
Superficial bleeding	1	Occurred at a trocar port site; treated with bedside sutures
Large epifascial hematoma	1	Surgically evacuated
Persistent pain	1	No obvious cause or consequences
Large subcutaneous hematoma	1	Treated conservatively
IPOM		
Widespread abdominal wall hematoma	1	Developed a widespread abdominal wall hematoma, requiring transfusion of two units of packed cells
Persistent pain re-laparoscopy	2	Persistent movement-dependent pain in the first few weeks; leading to relaparotomy and anchor sutures removal.
Abscess	1	Treated with incision and VAC.
Ongoing pain around anchor sutures	1	Persisted during follow-up; did not require further interventions
Subcutaneous hematoma and persisting pain	1	Prolonging the admission
Subcutaneous seroma	1	Developed subcutaneous seroma at a follow-up appointment

No statistically significant difference was observed regarding the severity of complications (*p* = 0,15).

Surgical site-specific complications were similar in both groups and no significant difference was observed in the rate of SSOs (eTEP: 5,43%, IPOM: 9,68%, *p* = 0,407), SSIs (eTEP: 1,09%, IPOM: 3,23%, *p* = 0,415) and SSOPIs (eTEP: 4,35%, IPOM: 3,23%, *p* = 0,784) (Table 5).

**Table 5:** Total group - Surgical site specific complications

Categories	eTEP (*n* = 92)	IPOM (*n* = 31)	*p*-value
SSO (%)	5 (5,43%)	3 (9,68%)	0,407
SSI (%)	1 (1,09%)	1 (3,23%)	0,415
SSOPI (%)	4 (4,35%)	1 (3,23%)	0,784

SSO = Surgical site occurrence; SSI = Surgical site infection; SSOPI = Surgical site occurrence requiring procedural intervention

A significant difference was observed in the median admission times between the two groups, with overall shorter hospital stays in the eTEP group (eTEP: 3 days [range: 1–12], IPOM: 4 days [range: 2–40], *p* < 0,001). Notably, there was an outlier in the IPOM group who was admitted for 40 days. Even after the outlier was removed, the difference between the two groups remained significant.

The postoperative outcomes are depicted in Table 3.

**Fig. 4:**
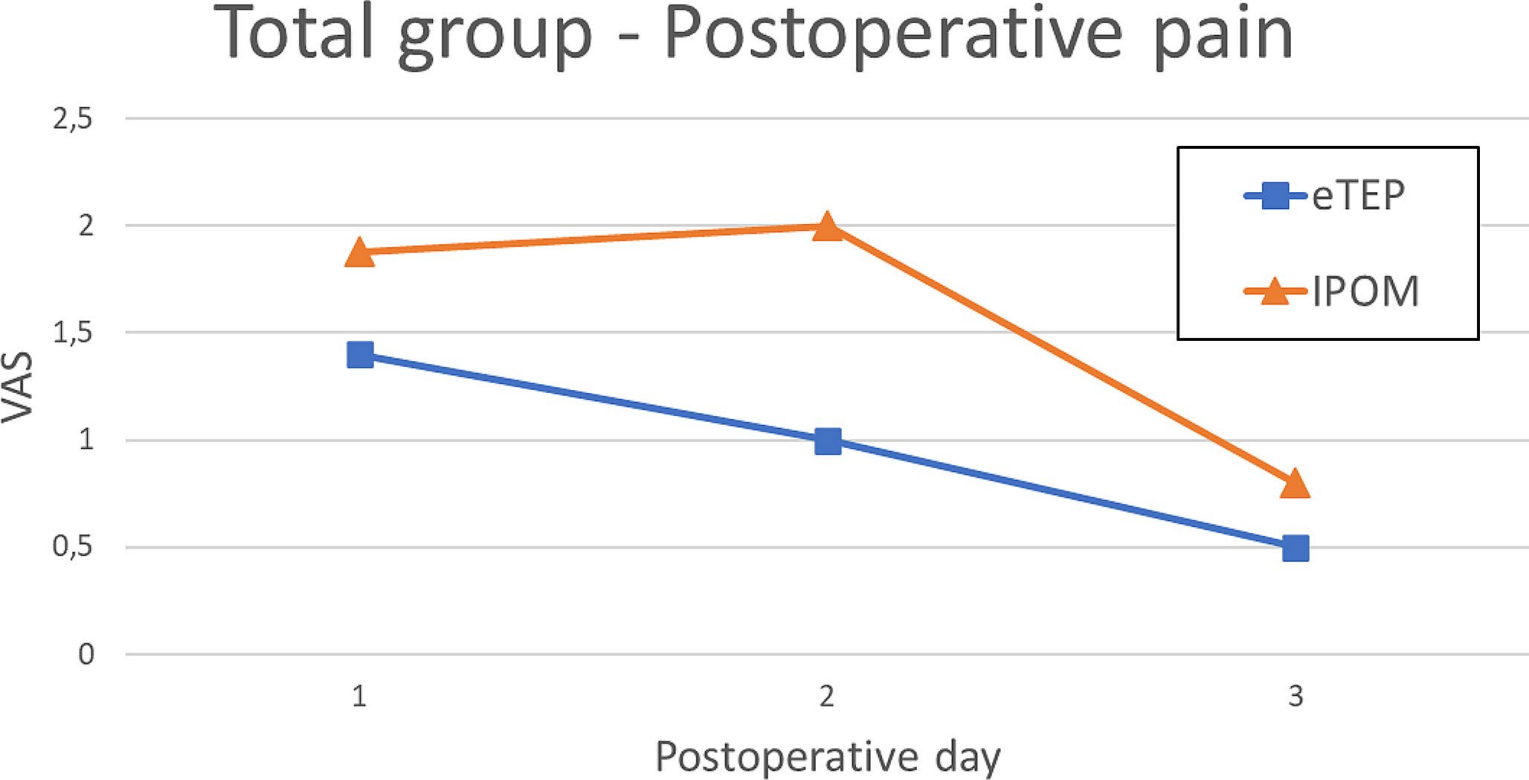
VAS for postoperative days 1–3 for the eTEP and IPOM group

No statistically significant difference was observed in average postoperative pain levels during the first three days, although the eTEP group generally reported lower pain levels than the IPOM group on each postoperative day (eTEP: first day: 1,4; second day: 1; third day: 0,5; IPOM: first day: 1,88; second day: 2; third day: 0,8; *p* = 0,438; *p* = 0,144; *p* = 0,929). Additionally, the eTEP group showed a quicker reduction in pain levels, starting on the second postoperative day. In contrast, the IPOM group experienced an increase in pain on the second postoperative day and improvement observed only on the third day (Fig. 4).

### Subgroup analysis

To enhance group homogeneity and account for the impact of previous surgeries as a potential confounder, a subgroup analysis was conducted exclusively on patients with primary hernias, including 72 patients from the eTEP group and 12 patients from the IPOM group. The outcomes of the subgroup analysis were comparable to the total group.

Overall, the eTEP and IPOM group with only primary hernias had a higher homogeneity. No significant differences in the demographics, risk factors and ASA scores were detected (Table 6).

**Table 6:** Subgroup analysis - demographics

Variables	eTEP (*n* = 72)	IPOM (*n* = 12)	*p*-value
Age (years)	51,31 (± 12,22)*	51,83 (± 18,73)*	0,899
BMI (kg/cm2)	31,96 (± 6,88)*	29,92 (± 6,01)*	0,335
Sex			0,306
Male (%)	48 (66,67%)	6 (50%)	
Female (%)	24 (33,33%)	6 (50%)	
ASA score			0,62
I	6 (8,33%)	2 (16,67%)	0,363
II	48 (66,67%)	7 (58,33%)	0,574
III	18 (25%)	3 (25%)	1
IV	0 (0%)	0 (0%)	/
V	0 (0%)	0 (0%)	/
Risk factors			
COPD (%)	2 (2,78%)	1 8,33(%)	0,337
Cardiovascular diseases (%)	35 (48,61%)	4 (33,33%)	0,326
Diabetes (%)	7 (9,72%)	1 (8,33%)	0,879
Smoking (%)	15 (20,83%)	5 (41,67%)	0,117
Liver cirrhosis (%)	0 (0%)	0 (0%)	/
Adipositas (%)	39 (54,17%)	5 (41,67%)	0,422
Defect size (cm2)	6 (IQR 9,13)†	4 (IQR 13)†	0,368

* = Mean + SD; † = Median + IQR; BMI = Body mass index; ASA = American Society of Anesthesiologists; COPD = Chronic obstructive pulmonary disease

The distribution of hernia types in the subgroup was in line with the total group analysis. There was a significant difference in the distribution of M3 hernias (*p* = 0,003), but not for any other hernia type (Table 7).

**Table 7:** Subgroup analysis - hernia types

European Hernia Society Classification	eTEP		IPOM		*p*-value
	*n* = 103	%	*n* = 12	%	
M1	2	1,94%	0	0%	0,559
M2	35	33,98%	6	50%	0,929
M3	62	60,19%	6	50%	**0**,**003**
M4	2	1,94%	0	0%	0,559
M5	2	1,94%	0	0%	0,559

Consistent with the total group analysis, significant differences were detected in the size of the mesh used (*p* < 0,001) and the admission period (*p* = 0,007).

In contrast to the total group, the subgroup had a significant shorter median operation time in the IPOM group (eTEP: 106,5 min. [range: 49–290], IPOM: 66,5 min. [range: 44–235], *p* = 0,043).

Similar to the total group, the subgroup analysis revealed a significantly higher rate of postoperative complications in the IPOM group (eTEP: 4,17%, IPOM: 25%, *p* = 0,009).

However, the complications were overall more severe in the eTEP group (*p* = 0.002). Most complications in the eTEP group were category IIIb (66.67%), while in the IPOM group, they were primarily category I (66.67%) according to the Clavien-Dindo classification.

Table 8 shows the postoperative outcomes of the subgroup analysis. Like the total group analysis, the subgroup analysis also did not reveal any significant difference in the rate of SSIs (eTEP: 1,39%, IPOM: 0%, *p* = 0,681), or SSOPIs (eTEP: 2,78%, IPOM: 0%, *p* = 0,559). However, a significantly higher rate of SSOs in the IPOM group was observed (eTEP: 2,78%, IPOM: 16,67%, *p* = 0,036) (Table 9).

**Table 8:** Subgroup analysis - outcomes

Variables	eTEP (*n* = 72)	IPOM (*n* = 12)	*p*-value
Mesh size (cm2)	450 (IQR 125)†	113 (IQR 187)†	**< 0**,**001**
Operation time (minutes)	106,5 (IQR 59)†	66,5 (IQR 72)†	**0**,**043**
Intraoperative complications ‡	0 (0%)	0 (0%)	0,461
Conversion Rate ‡	2 (2,7%)	0 (0%)	0,599
Postoperative complications	3 (4,17%)	3 (25%)	**0**,**009**
Clavien Dindo			**0**,**002**
I	0 (0%)	2 (66,67%)	
II	1 (33,33%)	1 (33,33%)	
IIIa	0 (0%)	0 (0%)	
IIIb	2 (66,67%)	0 (0%)	
IV	0 (0%)	0 (0%)	
V	0 (0%)	0 (0%)	
Admission period (days)	3 (IQR 1)†	4 (IQR 3)†	**0**,**007**
VAS			
Postoperative day 1	1,3 (IQR 1,7)†	2 (IQR 2,3)†	0,169
Postoperative day 2	1 (IQR 2)†	3 (IQR 3,15)†	0,102
Postoperative day3	0,25 (IQR 2)†	0,65 (IQR 2,25)†	0,86

* = Mean + SD; † = Median + IQR; ‡ = 74 cases were started as eTEP initially, but converted to IPOM intraoperative; VAS = Visual Analogue Scale

**Table 9:** Subgroup analysis - Surgical site specific complications

Categories	eTEP (*n* = 72)	IPOM (*n* = 12)	*p*-value
SSO (%)	2 (2,78%)	2 (16,67%)	**0**,**036**
SSI (%)	1 (1,39%)	0 (0%)	0,681
SSOPI (%)	2 (2,78%)	0 (0%)	0,559

SSO = Surgical site occurrence; SSI = Surgical site infection; SSOPI = Surgical site occurrence requiring procedural intervention

**Fig. 5:**
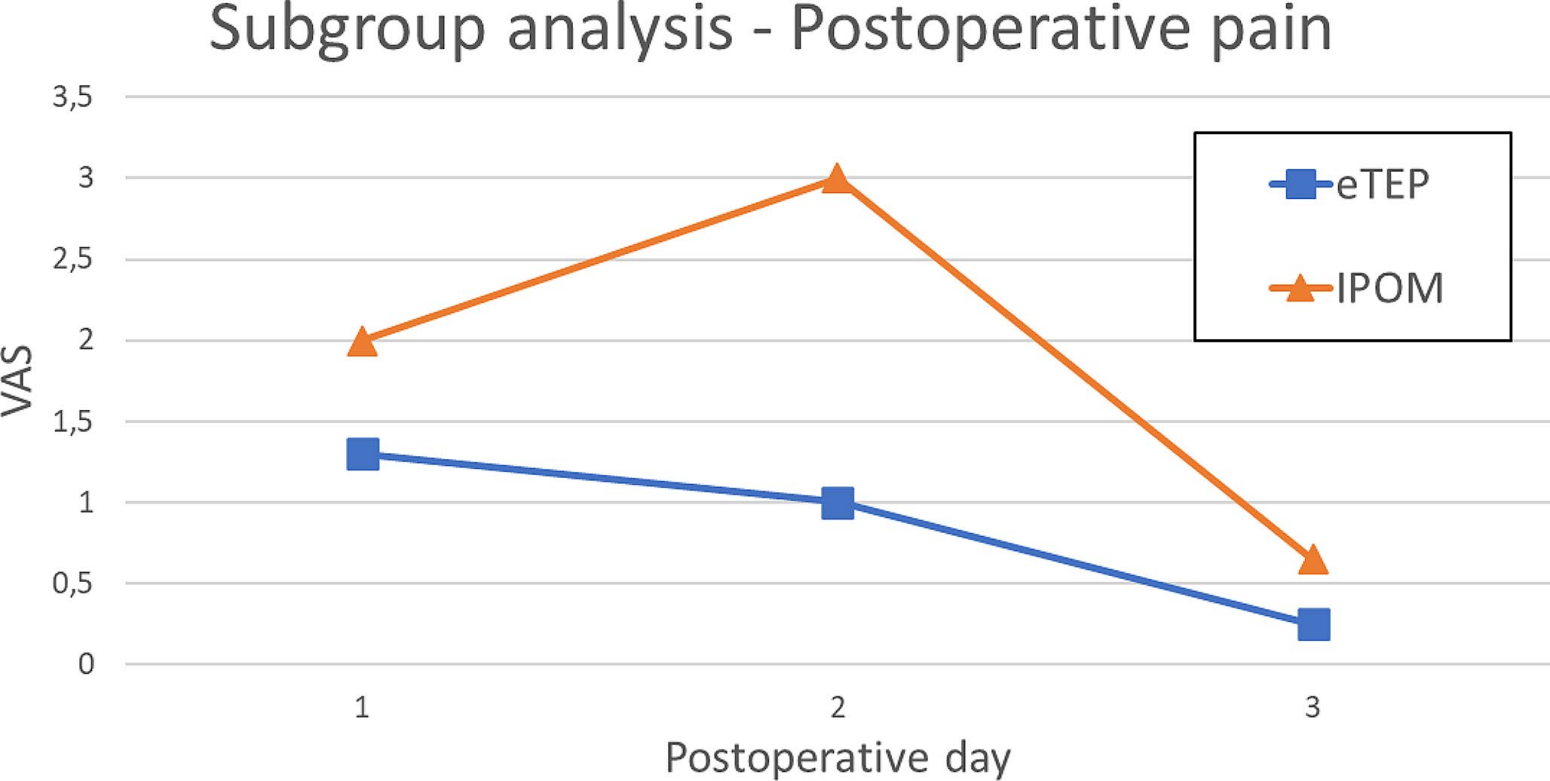
VAS for postoperative days 1–3 for the eTEP and IPOM group in the subgroup analysis

The postoperative pain was lower in the eTEP group, although without statistical significance. A slight increase in pain on postoperative day two was noted in the IPOM patients, similar to the trend in the total group analysis (Fig. 5).

## Discussion

Ventral hernias have always been a challenging surgery, and a wide number of surgical techniques have been developed for their optimal management. In recent years, minimal invasive procedures gained popularity, due to particular advantages over open approaches, including reduced risk infections and shorter hospital stays, as noted by the EHS [6].

However, it remains unclear which minimal invasive surgery is optimal in in terms of safety and feasibility. Consequently, the EHS advocates for further research in this area, particularly emphasizing the importance of studies aimed at comparing various treatment options. The pursuit of evidence-based recommendations remains a priority in the field, underscoring the importance of ongoing research and analysis to clarify the optimal approaches for ventral hernia management [6, 24].

The eTEP procedure is a promising surgical approach for ventral hernias. It builds on the principles of the Rives-Stoppa repair, which was first introduced in the 1960s [25]. Originally designed as an open repair method for inguinal hernias, the technique has since evolved to a minimal invasive procedure and was adapted for other hernia types as well [25].

There is a noticeable scarcity of literature on eTEP for ventral hernias, especially in Germany. To our knowledge, only one centre has reported its initial experiences with eTEP. Bauer et al. provided an analysis of 61 robotic-assisted eTEP procedures performed at their institution between 2019 and 2022 [26]. Given our larger database, we believe our study not only supplements their observations but also makes a substantial contribution to the existing body of evidence.

Our study aimed at comparing the intraoperative and early postoperative outcomes between eTEP and the more established IPOM procedure.

While some other studies have been conducted in this field, the literature remains limited. Most of the studies have relatively small sample size, a limitation that we addressed with our considerably larger dataset [10, 15–17]. Furthermore, our study distinguishes itself by performing a subgroup analysis of only primary hernias. Incisional hernias are associated with more challenging surgeries and can therefore act as significant confounder in the analysis of surgical outcomes [18, 19]. Excluding them in a subgroup analysis isolates this confounder, facilitating a clearer interpretation of the data. Except for one other study [15], the majority of the research we reviewed did not conduct such a subgroup analysis and incorporated the combined data from incisional and primary hernias into the analysis instead.

Overall, our results suggest that eTEP is a safe and feasible surgical procedure for treating ventral hernias, which is not inferior to the IPOM procedure and might even be superior in certain aspects.

After implementing eTEP in our centre, we observed a steep learning curve, reflected in decreased surgery time and expansion of our inclusion criteria. With rising experience, we started applying eTEP to more complicated hernias, including incisional hernias and those extending in the M1 or M5 area.

Initially, primary M1/M2 hernias were treated with a bottom-up approach, involving the suprasymphyseal placement of three trocars and caudal-to-cranial dissection of the retrorectus space. However, this technique was quickly abandoned since instruments collided frequently with the patients legs, creating difficult surgical conditions. Consequently, we switched to the classical eTEP approach for these hernias and performed a cross over in the epigastrium. If necessary, two periumbilical trocars were added to facilitate suturing in the epigastric area. In three cases, a TAR was needed for tension-free closure of the posterior fascia.

Previous research shows consistently shorter hospital stays for eTEP patients compared to IPOM. Confirming this trend, our study observed significantly shorter median admission times in the eTEP group (eTEP: 3 days [range: 1–12], IPOM: 4 days [range: 2–40], *p* < 0,001). The shorter admission times in the eTEP group were likely due to better pain management. Persistent or severe pain necessitated longer hospital stays. The difference in admission times might be attributed to more persistent pain in IPOM patients. Shorter hospital stays are desirable for cost savings, faster return to normal activities, and reduced risks of complications like deconditioning and infections. In this regard, eTEP appears to be superior [27, 28].

However, the theory that IPOM patients had less postoperative pain is not directly reflected by the VAS we measured during the first 3 days, as no significant difference was found. Nevertheless, average pain was lower in the eTEP group (eTEP: first day: 1,4; second day: 1; third day: 0,5; IPOM: first day: 1,88; second day: 2; third day: 0,8; *p* = 0,438; *p* = 0,144; *p* = 0,929). Furthermore, postoperative pain improved quicker in the eTEP group, with relief starting on the second day, compared to the third day in the IPOM group. The subgroup analysis showed similar trends, but without statistical significance.

Most other studies that compared the postoperative pain reported significantly lower pain scores in eTEP patients [10, 15–17]. The prevailing theory in the literature suggests that postoperative pain in eTEP patients is lower due to the lack of direct contact between the mesh and intestines, and the absence of anchor sutures, as the mesh is held in position by the rectus sheath [7, 9, 29].

It is unclear why our study did not replicate the significant postoperative pain findings reported by other researchers. One possible explanation is the large number of patients discharged on the second or third postoperative day. Without ambulatory pain monitoring, VAS scores were not recorded for these patients and thus not included in further data analysis. If patients with lower pain were discharged earlier and those with persistent pain stayed longer, the actual average postoperative pain might be lower than our findings suggest. This is notable given that the average admission period for the eTEP group was shorter than for the IPOM group. The potential overestimation of postoperative pain in the eTEP group, due to early discharges of patients with minimal pain, could have affected our ability to detect statistically significant differences. Additionally, all patients received analgesia according to our institutional protocol, which included a fixed opioids regimen for VAS of 4 or higher. This could have complicated especially the interpretation of higher pain values, by underestimating the pain of patients with higher VAS due to stronger analgesia. Therefore, the reported VAS should be interpreted with caution.

The total group analysis showed a shorter average operation time in the IPOM group, though without statistical significance (eTEP: 110 min, IPOM: 88 min, *p* = 0,064). However, the subgroup analysis revealed a statistically significant shorter average surgery time for IPOM patients (eTEP: 106,5 min., IPOM: 66,5 min., *p* = 0,032). Incisional hernias are typically more difficult to operate and require longer surgery times [18, 19]. Sine incisional hernias, which represented a significantly larger proportion in the IPOM group (IPOM: 61,29% vs. eTEP: 21,74%), were removed in the subgroup analysis, the results from the subgroup analysis are likely to be more accurate. Therefore, it can be assumed that eTEP procedures generally take more time than IPOM procedures. This aligns with the majority of previous studies [10, 16, 17, 30]. Prolonged surgery time is usually involved with higher costs and an increased risk of complications [31, 32]. Therefore, achieving shorter surgery times is desirable, making IPOM advantageous over eTEP in that regard.

We observed a decline in surgery time as the number of cases increased, likely reflecting our growing experience with eTEP. Similar observation were made by Bellido et al. and Bui et al. [10, 15]. Based on these findings, it could be assumed that the extended duration of eTEP surgeries partially relates to the procedure’s novelty and the surgeons initial lack of hands-on experience with it. A future analysis, after eTEP became even more of a routine procedure in our centre, might come to another conclusion and could be objective of future research.

The analysis revealed no statistically significant difference in the conversion rate between the eTEP and IPOM procedures (eTEP: 2,13%, IPOM 0%, *p* = 0,428). Nevertheless, two eTEP procedures, required conversion to IPOM. The first case involved a primary, 2 cm wide M3 hernia with rectus diastasis. The posterior rectus fascia was perforated while inserting the first trocar, hindering the expansion and dissection of the retrorectus space, necessitating a switch to IPOM. The second conversion occurred in a om a 2 cm wide M2 hernia, because the patient had previously been treated with a sublay mesh, which was not disclosed preoperatively. This resulted in adhesions in the retrorectus space, making expansion impossible. Since previous sublay mesh implantation is an exclusion criterion for eTEP, this patient should not have undergone eTEP, and thus, it is questionable whether it should be interpreted as a true conversion.

These conversions occurred during the early adoption of eTEP at our centre. In later cases, no conversions were necessary, reflecting our learning curve. Additionally, the lack of statistical significance and the smaller IPOM group suggest that the conversion rate difference is not necessarily a disadvantage of the eTEP procedure.

The rate of postoperative complications in the total group was significantly lower in the eTEP group (eTEP: 7,61%, IPOM: 22,58%, *p* = 0,023), the subgroup analysis was similar (eTEP: 4,17%, IPOM: 25%, *p* = 0,009). Due to the low number of total complications, analysing individual complication rates was not considered useful. However, when examining surgical site-specific complications, the subgroup analysis found a significant higher SSO rate in the IPOM group (*p* = 0,036). In the IPOM group, 2 SSOs occurred (one seroma and one postoperative bleeding), accounting for 16,67% of the cases, while in the eTEP group, 2 SSOs occurred (one postoperative bleeding and one early relapse), accounting for 2,28% of the cases. Similar findings were reported by Kudsi et al. [30]. However, since most other studies did not report significant differences in postoperative complication rates [10, 15, 17], and due to the fact the difference was only detected in the subgroup analysis, the relevance of these findings remains questionable.

When comparing the overall complications with other studies in this field, no obvious trend was observed, as complication rates and types had a wide variability between the centres and were generally too rare for effective statistical tests. However, the complications encountered for eTEP were consistent with those already known, particularly postoperative bleedings frequently reported in the literature [10, 15, 30].

While our findings suggest that eTEP procedures might have a lower risk of early postoperative complications, especially in terms of SSOs, further research, is necessary for a more comprehensive evaluation.

Although less frequent, postoperative complications in the eTEP group tended to be more severe in the subgroup analysis (*p* = 0,002). Two of the three complications in the eTEP group were classified as Clavien-Dindo IIIb (66,7%), (infection requiring VAC and an early hernia relapse requiring repeated surgery). In contrast all three complications in IPOM were either category I (postoperative seroma and persisting pain around the anchor sutures) or category II (bleeding requiring a transfusion). The total group analysis, however, did not detect any significant difference in the severity of complications (*p* = 0,15). Most other studies did not report a difference in the severity of postoperative complications [10, 15, 17] either, and one study even found a difference in favour of eTEP [30]. Therefore, the actual relevance of our remains questionable, especially due to the limited IPOM group size in the subgroup analysis.

This study focused on comparing early postoperative outcomes between eTEP and IPOM, so only complications which occured within the first 30 days were analysed. Therefore, no conclusions can be drawn about long-term outcomes, which will be the objective of future research.

Another objective for future studies will be the comparison of eTEP with other surgical techniques that utilise the retrorectus space, such as the open sublay technique, as this could provide further insights into the advantages and disadvantages of eTEP.

### Strengths and limitations

One of our study’s key strengths is the extensive dataset, which includes a substantial number of eTEP cases, enhancing the precision and accuracy of our findings. The subgroup analysis, excluding incisional hernias, provides a refined dataset, minimizing potential confounders. Additionally, all eTEP surgeries were performed by the same surgeon, ensuring consistency in technique and reducing variability in outcomes.

However, our study has limitations that must be considered for interpreting the results. The most significant is the size difference between the IPOM and eTEP groups, especially in the subgroup analysis. Due to the retrospective, non-randomized design and a decline in IPOM procedures at our clinic, equal group sizes could not be achieved. The statistical power remains limited, posing a risk for type 2 errors. Additionally, the retrospective nature of the study can lead to information bias, and in some cases, not all medical data could be retrieved.

## Conclusion

Based on our findings, eTEP appears to be a safe and feasible surgical treatment for ventral hernias. Compared to IPOM, it has the advantage of a shorter postoperative admission period but a longer operation time. Furthermore, our findings suggest a lower postoperative complication rate in eTEP. The postoperative pain might be lower in eTEP patients as well, however, our study could not demonstrate statistically significant findings. Further research is needed to confirm these findings and evaluate long-term outcomes.
